# Tension Pneumocephalus in a Tracheostomized, Chronically Ventilated, Duchenne’s Muscular Dystrophy Patient Without Prior Head Trauma

**DOI:** 10.7759/cureus.10389

**Published:** 2020-09-11

**Authors:** Vikas Kumar, Adedayo Oduwole, Albert Raminfard, Martin Barnes, Thuy-Hong Le

**Affiliations:** 1 Internal Medicine, Donald and Barbara Zucker School of Medicine at Hofstra/Northwell, Port Jefferson, USA; 2 Radiology, Stony Brook University Hospital/Mather Hospital, Port Jefferson, USA; 3 Internal Medicine, Donald and Barbara Zucker School of Medicine at Northwell/Mather, Port Jefferson, USA

**Keywords:** tension pneumocephalus, duchenne muscular dystrophy, case report

## Abstract

Tension pneumocephalus is a rare condition that can be a life-threatening neurosurgical emergency. It usually results from head trauma, but there have been case reports of iatrogenic causes including on non-invasive mechanical ventilation. We report a case of pneumocephalus resulting from high mechanical ventilation pressures in a patient without prior head trauma.

A 37-year-old male with Duchenne’s muscular dystrophy who had been ventilator-dependent through tracheostomy was admitted for shortness of breath and intermittent fevers. The patient was found to have pneumonia, with left-lower lobe consolidation, and was started on linezolid given known *Methicillin-resistant Staphylococcus aureus* from previous sputum culture; he was later switched to vancomycin and piperacillin-tazobactam given persistent fevers to cover for hospital-acquired pneumonia. The patient went into septic shock requiring multiple pressors as well as stress steroids for persistent shock, with eventual improvement in hemodynamics. He developed further respiratory acidosis on his usual ventilator settings, and peak inspiratory pressures (PIPs) progressively increased to as high as 45-70 cm H_2_O during his hospital course. PIPs did not improve with suctioning or after bronchoscopy. On the 17th day of the patient’s stay, he had acutely altered mental status with non-reactive fixed and dilated pupils and disconjugate gaze of the right eye on neurologic examination. CT of the head at that time revealed extensive pneumocephalus along the bifrontal convexities, suprasellar cisterns, and posterior fossa, with a possible fracture of the frontal skull base near the ethmoid roof. Mount Fuji sign was present on CT scan, indicative of “tension pneumocephalus”. Neurosurgical consultation was obtained but the family declined intervention given his overall debilitated stated. Comfort measures were instituted, and the patient expired the following day.

Pneumocephalus is the accumulation of air entry into the cranial cavity, generally from head trauma, inflammation, or surgery. Patients may have underlying base skull defects or microfractures that permit air to enter the intracranial cavity. Increased sphenoid sinus pressure from mechanical ventilation may enter the subperiosteal space, allowing air to enter the intracranial cavity. It is important to consider pneumocephalus in a patient with new neurological findings after mechanical ventilation.

## Introduction

Pneumocephalus is defined as a collection of air in the intracranial cavity [1]. It is a rare, typically benign condition, but can be a life-threatening neurosurgical emergency. Most frequently, pneumocephalus occurs after trauma or neurosurgical procedure [2]. The current literature on this condition is limited and predominated by case reports of iatrogenic causes of pneumocephalus, including mechanical ventilation [3]. We report a case of tension pneumocephalus resulting from high airway pressures during mechanical ventilation in a patient without prior head trauma or neurosurgical procedure.

## Case presentation

**Figure 1:**
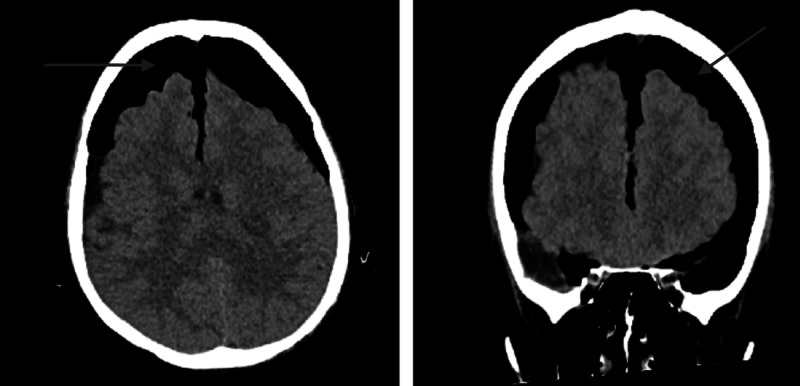
Non-contrast CT imaging in the axial (left) and coronal (right) views demonstrating the “Mount Fuji sign” for massive tension pneumocephalus. There is significant subdural air causing compression of the frontal lobes and subsequent compression away from the falx cerebri. The sulci are effaced, and ventricles have been compressed, suggestive of significant tension due to buildup of air pressure. [4]

A 34-year-old male with Duchenne’s muscular dystrophy and ventilator-dependent through tracheostomy presented with worsening shortness of breath, high fever, and altered mental status for one day. He was diagnosed with sepsis secondary to left lower lobe pneumonia and was started on empiric antibiotics. The patient was well known to our service, and due to his previous hospitalizations with sepsis due to pneumonia and *Methicillin-resistant Staphylococcus aureus* (MRSA) nasal colonization he was started on linezolid. Admission non-contrast computed tomography (CT) of his head revealed no acute intracranial process with mild frontal atrophy. The patient remained septic with persistent fevers despite appropriate treatment. He was switched to vancomycin and piperacillin-tazobactam after repeat respiratory cultures were positive for MRSA and Escherichia coli. A repeat head CT performed on day 14 for persistent sepsis and fluctuating mental status revealed no significant change. He continued to decline and developed septic shock requiring multiple vasopressors, fluid resuscitation, and empiric replacement dose steroids. Respiratory acidosis developed while on his usual ventilator settings with increasing peak inspiratory airway pressures (PIPs) to as high as 45-70 cm H_^2^_O. PIPs did not improve with suctioning or lowering tidal volume (from 450 cc to 350 cc); bronchoscopy showed no evidence of obstruction of the tracheostomy tube. Chest X-ray demonstrated worsening infiltrates, involving the entire left lung and areas of the right lung. On day 17, he became unresponsive with non-reactive fixed, dilated pupils, and disconjugate gaze of the right eye on the neurological examination. Repeat head CT at this point revealed extensive pneumocephalus along the bifrontal convexities, suprasellar cisterns, and posterior fossa. “Mount Fuji” sign was present (Figure 1), indicative of a tension pneumocephalus. Neurosurgical consultation was obtained, but the family declined intervention given his overall debilitated state. Comfort measures were instituted, and the patient expired the following day. An autopsy was declined.

## Discussion

Our chronically ventilated patient initially presented with sepsis secondary to pneumonia and altered mental status with no acute findings on head CT. During this hospitalization, he developed septic shock with respiratory acidosis requiring vasopressors and antibiotic therapy. The usual interventions used to reduce PIPs, including suctioning and reducing tidal volume, were ineffective, and PIPs remained high. He rapidly declined neurologically and was found to have a massive tension pneumocephalus. This occurred while being ventilated through tracheostomy and in the absence of head trauma or neurosurgical intervention. It is reported that pneumocephalus has an incidence of less than 1% in patients with head trauma [1]. Very few reports exist of patients developing pneumocephalus without skull fracture; however, these cases still report head trauma [1,5,6]. Pneumocephalus without prior head trauma or injury has not been reported. 

There are two major theories as to how pneumocephalus develops. The first is a mechanism described by Dandy called the Ball-and-Valve [7], which describes air trapping as it moves from the extracranial to intracranial space. The second is the Soda Bottle Theory by Horowitz, which postulates that Valsalva or loss of cerebrospinal fluid (CSF) causes a negative intracranial pressure, causing air to shift into the intracranial space [8]. Although the exact mechanism of pneumocephalus in our case is unknown, both mechanisms may have played a role. It is possible our patient had an underlying microfracture that was not detected on imaging; this would have allowed air to enter the intracranial space and become trapped under continued high positive pressure. 

Pneumocephalus is usually managed conservatively and rarely needs surgical correction. Supplemental oxygen increases the rate of air reabsorption [9] in spontaneously breathing patients, but avoidance of positive pressure is essential. This condition is managed by correcting the underlying cause, correcting the basal skull fracture causing continued air entry, or stopping continued CSF leak. When significant or sudden neurological deficit is present, emergent neuroimaging and neurosurgical evaluation may potentially be lifesaving.

## Conclusions

Tension pneumocephalus is a very rare condition usually associated with head trauma or neurosurgical intervention. Although it is uncommon in patients on positive pressure ventilation, it is important to consider. Despite all efforts, high PIPs were unavoidable in our case and resulted in the rare complication of tension pneumocephalus.
